# Marine Actinomycetes: A New Source of Compounds against the Human Malaria Parasite

**DOI:** 10.1371/journal.pone.0002335

**Published:** 2008-06-04

**Authors:** Jacques Prudhomme, Eric McDaniel, Nadia Ponts, Stéphane Bertani, William Fenical, Paul Jensen, Karine Le Roch

**Affiliations:** 1 Department of Cell Biology and Neuroscience, University of California Riverside, Riverside, California, United States of America; 2 Department of Biochemistry, University of California Riverside, Riverside, California, United States of America; 3 Center for Marine Biotechnology and Biomedicine, Scripps Institution of Oceanography, University of California San Diego, San Diego, California, United States of America; University of California Los Angeles, United States of America

## Abstract

**Background:**

Malaria continues to be a devastating parasitic disease that causes the death of 2 million individuals annually. The increase in multi-drug resistance together with the absence of an efficient vaccine hastens the need for speedy and comprehensive antimalarial drug discovery and development. Throughout history, traditional herbal remedies or natural products have been a reliable source of antimalarial agents, e.g. quinine and artemisinin. Today, one emerging source of small molecule drug leads is the world's oceans. Included among the source of marine natural products are marine microorganisms such as the recently described actinomycete. Members of the genus *Salinispora* have yielded a wealth of new secondary metabolites including salinosporamide A, a molecule currently advancing through clinical trials as an anticancer agent. Because of the biological activity of metabolites being isolated from marine microorganisms, our group became interested in exploring the potential efficacy of these compounds against the malaria parasite.

**Methods:**

We screened 80 bacterial crude extracts for their activity against malaria growth. We established that the pure compound, salinosporamide A, produced by the marine actinomycete, *Salinispora tropica,* shows strong inhibitory activity against the erythrocytic stages of the parasite cycle. Biochemical experiments support the likely inhibition of the parasite 20S proteasome. Crystal structure modeling of salinosporamide A and the parasite catalytic 20S subunit further confirm this hypothesis. Ultimately we showed that salinosporamide A protected mice against deadly malaria infection when administered at an extremely low dosage.

**Conclusion:**

These findings underline the potential of secondary metabolites, derived from marine microorganisms, to inhibit *Plasmodium* growth. More specifically, we highlight the effect of proteasome inhibitors such as salinosporamide A on *in vitro* and *in vivo* parasite development. Salinosporamide A (NPI-0052) now being advanced to phase I trials for the treatment of refractory multiple myeloma will need to be further explored to evaluate the safety profile for its use against malaria.

## Introduction

Throughout history, secondary metabolites (natural products) have provided a fundamental source of drugs for fighting infection, inflammation and cancer in humans. In the case of malaria, leveraging biodiversity in the natural environment has been one of the most effective ways of combating the disease. Quinine was extracted from the bark of a Peruvian *Cinchona* tree more than 350 years ago. It has been the most widely used drug until 1944 at which time it was replace by chloroquine that was successfully synthesized in 1934. The herb *Artemisia annua*, utilized by the Chinese to cure malaria for more than 2000 years, is the source of the antimalarial, artemisinin. Despite intense research, there is no doubt that plant-derived compounds have outlived many of the synthetic drugs.

Although artemisinin provides efficacy, recent studies have detected the emergence of drug resistance in the rodent malaria, *Plasmodium yoelii* [1], [2]. Drug resistances in the human malaria parasite, *P. falciparum,* were also observed *in vitro* [*3*] *.* Recently, artemisinin combinatorial therapy failures have been observed in Cambodian patients [4]. Because of the constant emergence of resistant strains and the absence of effective vaccines, there is a pressing need to rapidly discover new cost-effective molecules against the malaria parasite, which continues to kill 1.5 to 3 million people each year.

Constituting more than 70% of the earth's surface, our oceans emerge as one of the greatest sources of biodiversity for the discovery of natural products. Marine plants and invertebrates have received much attention as a source of human therapeutics. For example, a number of kinase inhibitors such as hymenialdisine (HMD), a sponge-derived natural product with nanomolar activity against several human kinases, have garnered much attention from pharmaceutical companies [5]. Today several marine-derived compounds used as anticancer agents are undergoing promising preclinical and clinical development [6]. Although no marine natural products have yet been approved for antimalarial use, the malaria research community has a long-standing interest in assessing marine derived compounds as new chemotherapies against malaria. Molecules such as hymenialdisine and xestoquinone, both extracted from marine sponges, strongly inhibit *Plasmodium* growth and *Plasmodium*-derived kinases [7], [8], [9], [10]. These promising results have led to the conclusion that kinase inhibitors represent a suitable chemotherapy against the malaria parasite. Another promising antimalarial agent derived from a marine sponge is manzamine A [11]. It has been proposed that the true source of this alkaloid is a symbiotic bacterium living within the sponge tissues (United States Patent Application 20050244938), thus providing opportunities for a renewable supply of the compound without harvesting large quantities of the sponge from nature.

Today, the microorganisms residing in deep ocean sediments remain an untapped resource for natural compound discovery. Included among these microorganisms are actinomycetes, a group of bacteria that accounts for more than 50% of the antibiotics identified to date [12]. These sediment-derived actinomycetes include obligate marine taxa [13], and an impressive array of readily cultivable diversity that has not been previously reported from land [14]. In the past few years, chemical studies on marine actinomycetes have yielded numerous novel secondary metabolites that display a wide range of activity against human tumors and other disease targets [15]. However, to our knowledge, none of these compounds have been tested against parasitic diseases. We therefore decided to test the ability of extracts from marine sediment-derived actinomycetes to inhibit the human malaria parasite, *P. falciparum*.

Our laboratory employed an adapted SYBR Green assay in microplate format to screen a set of crude extracts from marine microorganisms for their ability to inhibit parasite growth in culture. One extract from the marine actinomycete, *Salinispora tropica,* was selected for its high potency against parasite growth. Pure active compounds from *Salinispora tropica* had been previously identified and exhibited inhibitory effects in many human malignant cell types [16], [17], [18], [19]. Salinosporamide A, was identified as a potent inhibitor of dividing melanoma cells and showed a unique ability to inhibit the proteolytic activity of the 20S proteasome subunit without affecting any other proteases [20], [21]. The encouraging preclinical properties of this compound and its entry into clinical trials phase I (Nereus Pharmaceuticals) [22] motivated us to investigate further this orally active proteasome inhibitor against *Plasmodium.* We first tested the effect of pure salinosporamide A on parasite culture *in vitro*. The compound showed strong growth inhibition with an IC_50_ in the low sub-micromolar range (11.4 nM). At the phenotypic level, we identified an arrest of the parasite's progression during all erythrocytic stages. In addition, we detected an increase of ubiquitin conjugate proteins in parasite extracts after incubation with the drug. Similar results were obtained with the well-defined proteasome inhibitor MG-132 and lead us to conclude that the parasite proteasome was the likely target of salinosporamide A. Sequence comparisons and crystal structure modeling between salinosporamide A and yeast, human and *Plasmodium* 20S proteasome subunits further confirmed this potential target in the parasite. We then defined the efficiency of the compound in a malaria mouse model and found that the compound inhibited parasite growth and cleared parasitemia in treated mice at extremely low doses (130 µg/kg). Through this process, we have identified that salinosporamide A represents a new class of antimalarial drugs, which have the benefit of already proceeding to phase I clinical trial.

## Results

### Screening inhibitors by parasite proliferation assays in P. falciparum

**Figure 1:**
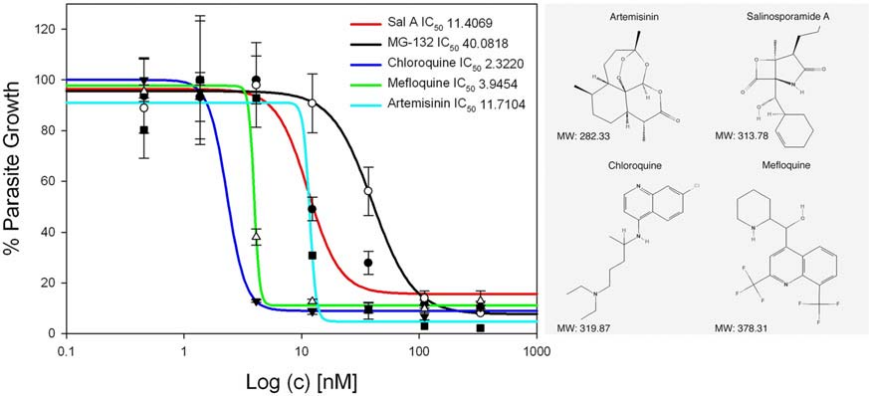
Inhibition of *P. falciparum* growth by proteasome inhibitors and standard antimalarial drugs with their chemical structures. IC_50_ values of parasite treated with the drug were determined using the SYBR Green assay and calculated by non linear regression using four-parameter logistic curves on SigmaPlot 10.0 software (values are mean±standard error of the mean). Each value in the curve is the average of 2 different experiments±standard deviation. Salinosporamide A inhibited the proliferation of the parasite at a low nanomolar range (IC_50_ = 11.4±1.9 nM) suggesting that the compound is as efficient as standard antimalarials (e.g. chloroquine (2.3±0.15 nM), mefloquine (3.9±2.7 nM) and artemisinin (11.7±8.7 nM). The inhibitor MG-132 is also a potent inhibitor of the parasites growth *in vitro* (IC_50_ of 40±4.8 nM) suggesting a strong inhibition effect of general proteasome inhibitors. Correlation coefficients (*R* values-factor of regression calculated by SigmaPlot 10.0) were 0.9887 for Sal A, 0.9996 for chloroquine, 0.9989 for mefloquine, 0.9900 for artemisinin and 0.9962 for MG-132.

Parasite proliferation assays were done in 96 well plates using the DNA intercalating fluorescent dye, SYBR Green [23], [24]. Using this technique, we measured the increase of parasite DNA contained in human red blood cells after 72 hours of incubation with our microbial extracts. Extracts were tested at three initial concentrations: 1 µg/ml, 10 µg/ml and 100 µg/ml. Five crude extracts showed a 50 to 100% inhibition effect at 1 µg/ml per ml (Data not shown). The most active fraction was further selected and identified to be the marine actinomycete, *Salinispora tropica*. The most active compound of *S. tropica*, salinosporamide A, had previously exhibited significant cytotoxic activity against human tumor cells lines [16], [17], [18], [19]. We then further tested the *in vitro* effect of salinosporamide A, which showed an IC_50_ value against parasite growth at 11.4±1.9 nM (Figure 1). This result falls within the previously reported IC_50_ range of chloroquine, mefloquine and artemisinin under our culture conditions (Figure 1). These initial results validate our assay and the efficacy of this new natural compound against *Plasmodium in vitro*.

In order to establish a baseline for proteasome inhibition and the effect on parasite growth, we tested the commercially available inhibitor MG-132 and found that this specific proteasome inhibitor arrests parasite growth with an IC_50_ value of 40±4.8 nM (Figure 1).

### Parasite phenotypic analysis in presence of salinosporamide A

**Figure 2:**
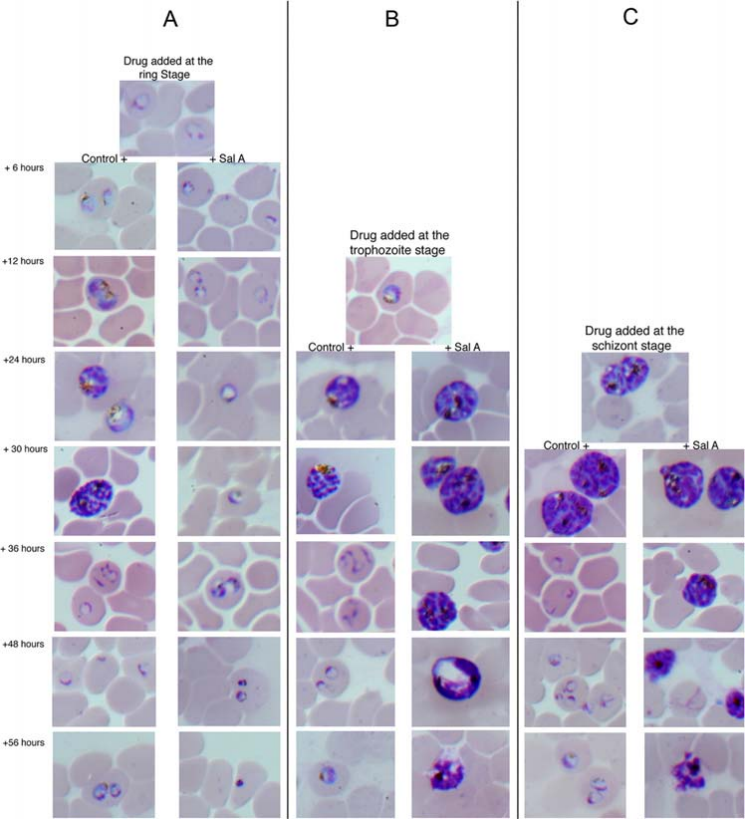
Effect of salinosporamide A on parasite morphology. Parasites were synchronized twice using sorbitol method. Salinosporamide A was added at the IC_80_ to ring (A), trophozoite (B) or schizont (C) stage. Morphological changes were observed every 6 or 12 hours by microscopic examination.

In order to investigate the most efficient erythrocytic stage of action of salinosporamide A against the parasite cell cycle, we proceeded to a phenotypic analysis of the treated and non-treated cultures at the three main stages of the parasite intra-erythrocytic cycle. Parasites were synchronized using sorbitol treatment and then incubated with the IC_80_ concentration (120 nM) of salinosporamide A at the ring, trophozoite and/or schizont stages. Parasites phenotypes were analyzed under light microscopy every 6 or 12 hours. After 6 hours in the ring stage of incubation with the drug, parasites were clearly arrested in their cell cycle progression when compared to the untreated cultures (Figure 2A). After 36 to 48 hours of incubation, parasite incubated with the drug showed signs of stress with no reinvasion into red blood cells while untreated culture shows rings reemerging with a 10 fold increase in parasitemia. When the drug was added at the trophozoite stage, parasites were arrested at a late trophozoite stage and showed signs of stress and a decrease in parasitemia after 36 hours (Figure 2B). When the drug was added at the schizont stage, the cell cycle progression was rapidly arrested. No parasite rupture of red blood cells could be detected and schizonts expired within a few hours (Figure 2C). These results demonstrate that salinosporamide A is an extremely efficient inhibitor during the erythrocytic stages of the malaria parasite.

### Biochemical analysis of the effect of salinosporamide A in parasite extracts

**Figure 3:**
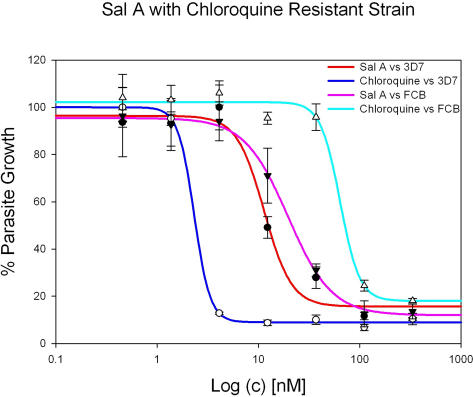
Inhibition effect of salinosporamide A on chloroquine resistant *P. falciparum* strain FCB. IC_50_ values of parasite treated with the drug were determined using the SYBR Green assay. Each value in the curve is the average of 2 different experiments±standard deviation. Salinosporamide A inhibited the proliferation of the FCB strain suggesting that the compound is equally active against drug resistant parasites. IC_50_ values with Salinosporamide A were 11.4 nM±1.9 for 3D7 (*R* = 0.9887) and 19.6 nM±1.4 for FCB (*R* = 0.9990). Chloroquine IC_50_ values were 2.3 nM±0.15 (*R* = 0.9996) and 64.1 nM±4.7 (*R* = 0.9964) for 3D7 and FCB respectively.

To investigate the potential mode of action of salinosporamide A in the malaria parasite, we examined the amount of ubiquitinated protein conjugates in parasite extracts after 6 hours of incubation with the drug. As shown in figure 3, using an anti-ubiquitin antibody, we demonstrate a considerable increase of ubiquitinated protein when incubated with the drug. We obtained a comparable increased of protein ubiquitination with the proteasome inhibitor MG-132 when compared to the untreated parasite cultures. Taken together, these data show that both of these drugs inhibit the degradation of ubiquitin-tagged target proteins and that the inhibition of ubiquitin conjugate degradation by the proteasome may be responsible for the cell cycle arrest detected by the analysis of the parasite morphologies under light microscopy. The data presented here show that salinosporamide A may certainly target a major player in the cell cycle control in eukaryotic cells: the proteasome.

### Activity against resistant P. falciparum strain

**Figure 4:**
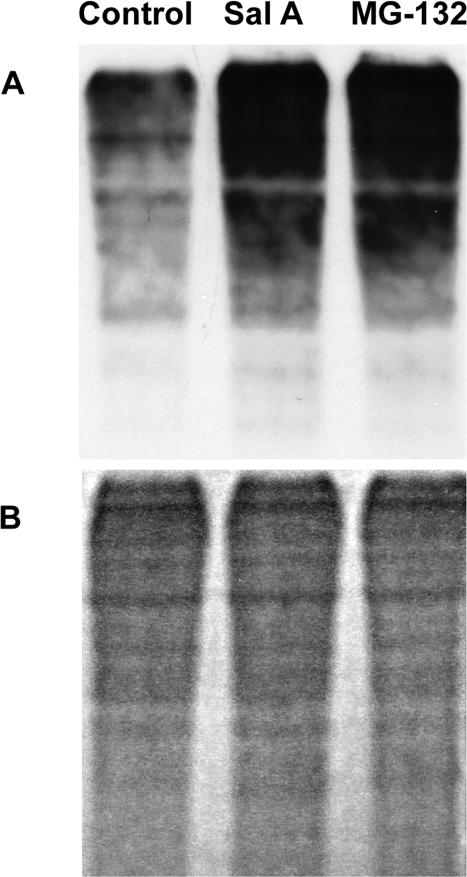
Western blot analysis of parasite proteins using anti-ubiquitin antibodies. Synchronized 3D7 parasite cultures were treated with the IC_80_ value of salinosporamide A (line 2) or MG-132 (Line 3). Following parasite treatment with the drug, parasite extracts were analyzed for the presence of ubiquitin-conjugates. Ubiquitinated proteins accumulate in the drugs treated when compared to the untreated cultures.

In order to confirm the efficacy of salinosporamide A on divergent parasite strains, we tested the compound on a resistant *P. falciparum* strain. We examined the FCB chloroquine resistant strain and confirmed the effectiveness of salinosporamide A against drug sensitive and drug resistant parasites (Figure 4).

### Homology modeling and virtual ligand interaction

**Figure 5:**
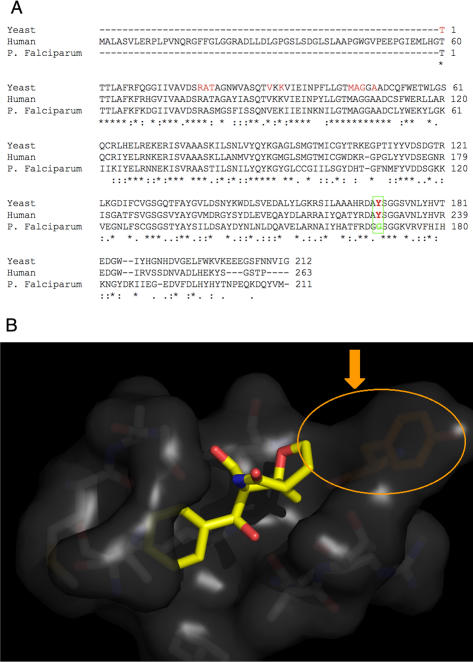
(A) Sequence alignment of the catalytic domain of the β5 subunit 20 S proteasome from yeast, human and *Plasmodium* obtained with the ClustalW program. (B) Crystal structure of Salinosporamide A interacting with the yeast 20S proteasome. Tyr168 is shown in orange to indicate the site of the Y168G mutation in *P. falciparum.*

In an attempt to investigate the structure relationship of salinosporamide A with the parasite's proteasome, we examined the published crystal structures of the yeast 20S proteasome core particle in complex with salinosporamide A [25]. We identified the amino acids of the core proteasome interacting with the compound (Figure 5 A-amino acids highlighted in red). Using ClustalW, we aligned and compared yeast, human and *Plasmodium* beta5 subunit proteasome sequences. The catalytic domain was extremely well conserved except for a single mutation (Y168G) between human and *Plasmodium* sequences (Figure 5 A- Amino Acid highlighted in green). Taken together, these data show that the parasite proteasome is likely the target of salinosporamide A in *Plasmodium*.

### Activity against rodent parasites

**Figure 6:**
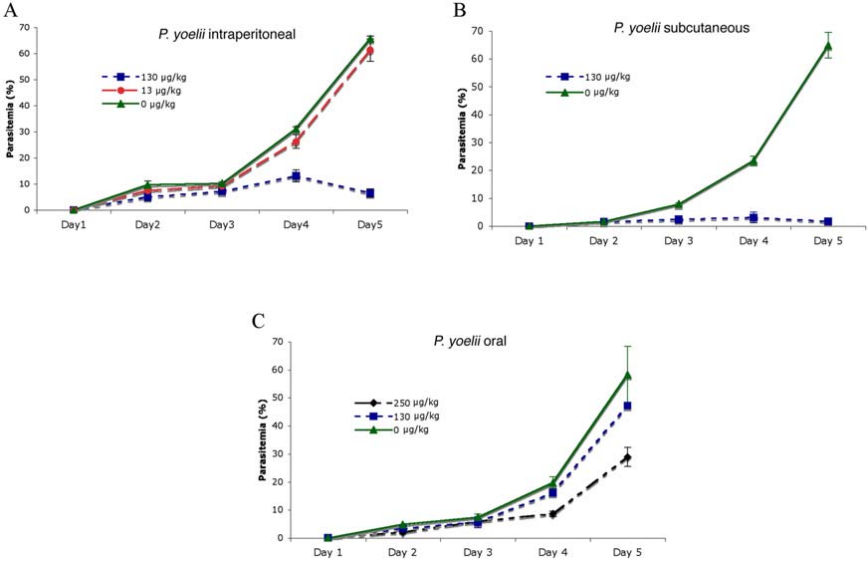
In vivo activity of salinosporamide A against Rodent malaria parasite, *P. yoellii*. Mice were treated using 3 independent delivery methods: A) Intraperitoneal B) Oral Route and C) Subcutaneous. Mice treated by oral route at 250 µg/kg (*t*-test, *p* = 0.062) or by intra peritoneal and subcutaneous methods at 130 µg/kg showed a significant decrease of parasitemia when compared with control mice (*p*<0.001). Parasitemia was almost cleared when used at 130 µg/kg when delivered by subcutaneous method (*p*<0.001). Values are means±standard deviation.

Based on our positive *in vitro* results and the favorable preclinical properties of salinosporamide A in mice against human lymphoma [22] we decided to test the *in vivo* efficacy of this drug using the parasite mouse model, *P. yoelii yoelii .* The studies were carried out on eight groups of five mice. *P. yoelii* was injected in mice at day one. If viable parasites were detected in the blood smear at day two, salinosporamide A was injected IP at two different concentrations (13 µg/kg and 130 µg/kg) on days 2, 3 and 4. For each group, untreated and treated mice were bled each day from the tail to evaluate the percentage of infected red blood cells. Percentage of parasitemia was monitored by microscopic observation of fixed giemsa stained blood smears and further confirmed by an independent scientist using flow cytometry analysis (See Materials and Methods for further details). The study was terminated on day 5 when control mice had reached a high parasitemia in the blood (above 60%). Our results clearly show a rapid and significant decrease (t-test, p = 7.9 E-04) of the parasitemia (5.5 fold decreased) when treated at 130 µg/kg (Figure 6A). We then wanted to test the efficacy of Salinosporamide A when injected subcutaneously at one single concentration (130 µg/kg) of dose on day 1, 2 and 4. Using this method, parasites never managed to replicate actively when compared to the control group (t-test, p = 1.9 E-05) (Figure 6B). To further examine the oral activity of Salinosporamide A, infected mice were treated by oral route at 130 µg/kg and 250 µg/kg on day 1, 2 and 4 (Figure 6C). At 130 µg/kg no significant decrease was observed under our experimental conditions. However at 250 µg/kg, a two-fold parasitemia decrease was observed when compared to the control group (t-test, p = 0.06). Using the three independent administration methods, we show that Salinosporamide A can be considerably effective *in vivo.*

## Discussion

Marine actinomycetes are recognized as a rich source of secondary metabolites with activities against cancer. In this study, we have shown that extracts from ocean sediment derived microorganisms also provide a good source for the discovery of small molecules with antimalarial activity. We have demonstrated that the pure compound salinosporamide A, isolated from the marine actinomycete *Salinispora tropica,* is a highly potent inhibitor of the human malaria parasite *in vitro* and *in vivo*. Salinosporamide A, related to the known proteasome inhibitor omuralide [26], was later shown to be a potent inhibitor against human multiple myeloma [22]. The specific inhibition was due to the ability of the atypical fused δ-lactam-β-lactone ring structure of salinosporamide A to bind covalently to the active threonine site of the 20 S proteasome subunit [16], [27].

The ubiquitin/proteasome system (UPS) is known to mediate intracellular protein degradation for misfolded, damaged and key regulatory proteins involved in various essential cellular functions such as transcription, stress response, cell cycle, cell differentiation and DNA repair [28]. The UPS involves the labeling of key target proteins with a small regulatory protein, ubiquitin, via a cascade of three enzymes termed E1, ubiquitin-activating enzyme; E2, ubiquitin-conjugating enzyme; and E3, ubiquitin-ligase. Once poly-ubiquitinated, target proteins are degraded by the proteasome complex. The importance of the UPS has been widely illustrated by the fact that protein abnormalities in this pathway are implicated in the pathogenesis of many human diseases, including cancer and neurodegenerative diseases such as Alzheimers or Parkinsons. Targeting the proteasome system has a huge therapeutic implication as it can restrain growth and survival of most cell types. The limited toxicity of proteasome inhibitors results from the fact that rapidly dividing cells are more sensitive to the inhibitor than non-dividing cells. Recent pre-clinical and clinical studies validated the 20S proteasome as an excellent therapeutic target. The outcome of these studies resulted in the FDA approval of the first proteasome inhibitor, Bortezomib (Velcade®) for the treatment of refractory multiple myeloma in 2003 [29]. Comparative genomic analysis against 13 different eukaryotic genomes identified all components of this system in apicomplexan parasites including five *Plasmodium* species (Ponts N. et al. 2008 Manuscript submitted). Partially conserved sequences of the proteasome subunits across species, as well as the exponential growth of the parasite in red blood cells, suggest that the proteasome is the probable target of salinosporamide A in the malaria parasite. Its inhibitory effect with an IC_50_ of 11.4 nM is as efficient as the majority of conventional antimalarials such as chloroquine or artemisinin (Figure 1). It is also interesting to note that chloroquine resistant strains are still sensitive to salinosporamide A (Figure 4). Previous studies have demonstrated development and growth inhibition by proteasome inhibitors such as lactacystin or bortezomib related compounds (MLN-273) on diverse apicomplexan parasites at different stages of their life cycle [30], [31], [32], [33], [34]. The IC_50_ value of 40 nM of the well-defined proteasome inhibitor MG-132 only strengthens the validity that proteasome inhibitors are efficient against parasite growth *in vitro*. In eukaryotic cells, the proteasome is known to control key regulatory proteins involved in G1/S and M checkpoints of the cell cycle. Progression of the erythrocytic cell cycle in *Plasmodium* is poorly understood. Several key regulatory proteins such as CDK/cyclin, which are known to be targets of the proteasome in eukaryotes, have been identified and characterized in *Plasmodium* but the mechanisms by which they are regulated, are largely unknown. Microscopic analysis of treated *P. falciparum* clearly showed an arrest of the cell cycle progression at the ring and schizont stage before DNA synthesis and at the time parasites rupture their red blood cells, respectively. These effects were observed with lactacystin treatment and corroborate with the parasite proteasome to control the erythrocytic cycle progression before and after the DNA synthesis (S phase). The exact mode of action by which salinosporamide A inhibits *Plasmodium* erythrocytic development is not known. However, the increased detection of parasite ubiquitin protein conjugates after 6 hours of incubation with the drug when compared to untreated cultures validates the likely inhibition of the *Plasmodium* proteasome complex. It is therefore expected that the inhibition effect of salinosporamide A results from the regulation of key cell cycle proteins within the parasite. In addition, comparative proteasome sequence analysis and crystal structure modeling further validate the parasite proteasome as the potential target.

In agreement with the *in vitro* data, salinosporamide A inhibits parasite growth *in vivo* at a very low dose (130 µg/kg). This result has been validated using three independent modes of drug administration in separate groups of mice. Assessment of the drug administration in mice or other model organisms will need to be developed more carefully. This orally active compound is now being evaluated for phase I trials at Nereus Pharmaceuticals for the treatment of refractory multiple myeloma. Dosage is in the 150 to 500 µg/kg range. The safety profile of this compound and its appropriate formulation will need to be further evaluated for its potential to treat malaria.

### Conclusions

Today, the urgent need for new antimalarials requires the discovery of small and inexpensive molecules. Using a SYBR Green bioassay on the parasite's erythrocytic stages we have determined that a secondary metabolite produced by a marine bacterium has significant antimalarial activity. This finding demonstrates that natural products remain one of the most important sources of medicines against the parasite. The pure compound, salinosporamide A, was tested for its inhibitory activity against parasite development *in vitro (P. falciparum)* and *in vivo (P. yoelii*). Our biochemical and structural-based analyses are consistent with the parasite 20S proteasome being the molecular target. The divergence observed at the structural level facilitates the discovery of an increased specificity of *Plasmodium* proteasome inhibitors. We are now pursuing a structure-activity relationship analysis to design a more potent and selective malarial proteasome inhibitor.

## Materials and Methods

### Preparation of the natural product extracts and pure compound

Eight strains of diverse, Gram-positive marine bacteria were grown in 5 liter culture, extracted with ethyl acetate, and the extracts concentrated and separated into nine fractions by normal phase silica chromatography using a solvent gradient from 100% iso-octane to 10% methanol in ethyl acetate. Each crude extract and corresponding set of nine fractions (10 samples total per strain) were concentrated to dryness, weighed, brought to a standard concentration of 25 mg/ml in DMSO, and dispensed (10 µl) into 96-well microtiter plates. Salinosporamide A was purified from a large-scale culture of *Salinispora tropica* strain CNB-392 as previously described [16].

### Cultivation of *P. falciparum*

*P. falciparum* malaria parasites (3D7 and FCB strains MR4/ATCC, Manassas,VA) were cultured in human type O+ erythrocytes as previously described [35]. Synchrony was achieved through sorbitol lysis of mature forms [36].

### SYBR Green based parasite proliferation assay

Compounds were diluted in complete medium and 40 µl transferred to 96-well assay plates (Costar #3094, Corning, NY). To this solution, 80 µl of complete media with 3D7 infected erythrocytes were dispensed at 2.5% hematocrit and 0.5% parasitemia in the assay. Uninfected erythrocytes were dispensed into the background wells at the same final hematocrit. Plates were incubated for 72 hours in a low oxygen environment (96% N_2_, 3% CO_2_, 1% O_2_) in a modular incubation chamber (Billups-Rothenberg, Del Mar, CA). The plates were sealed and placed in a −80°C freezer overnight then thawed and 120 µl of lysis buffer (20 mM Tris-HCl, pH 7.5, 5 mM EDTA, 0.08% Triton X-100, (Promega), 0.008% saponin (Acros)) with 0.2 µl/ml Sybr Green I (Invitrogen) was dispensed into each well and incubated at 37°C in the dark for 6 hours to achieve an optimum signal to noise ratio. The plates were read with a Molecular Devices SpectraMAX Gemini EM at ex: 495 nm, em: 525 nm with 515 nm cut-off.

### Microscopic analysis

Thin smears of malaria culture were stained with giemsa and observed with a Nikon Eclipse E200 microscope. Images were captured with by a Cannon EOS Rebel XT camera mounted on an Olympus Bx51 microscope through a 60x/1.4 N.A. oil objective utilizing Digital Photo Professional software. (Courtesy of Morris Maduro lab.)

### Western blot analysis

Synchronized cultures were grown in parallel in the presence of 140 nM salinosporamide A, 400 nM MG-132 (Calbiochem) or no drug (control) added at the mid-trophozoite stage. After 8 hours incubation cultures were collected, washed with PBS and the parasites extracted by 0.15% saponin lysis (Acros). Subsequent washes were performed in PBS supplemented with 20 mM NEM (Pierce), 0.05 mM EDTA (Promega), 1 mM AEBSF (Fisher Bioreagents), 0.02% Sodium Azide and Protease Inhibitor Cocktail (Complete, Roche). Proteins were extracted from parasite pellets through sonication in lysis buffer (50 mM TRIS-HCl, pH 7.5 (Promega), 150 mM NaCl, 2 mM AEBSF (Fisher), 20 mM NEM (Pierce), 0.5 mM EDTA, 1% Triton X-100 (Promega), Protease Inhibitor Cocktail (Complete, Roche), 0.02% Na Azide). Proteins were separated by SDSPAGE with a BioRad mini PROTEAN 3 cell and transferred to Invitrogen PVDF membranes with an Owl Panther semidry electroblotter. Blots were probed with Upstate anti-ubiquitin polyclonal antibody and goat anti-mouse HRP secondary (Pierce) and visualized with Amersham ECL western blotting substrate.

### Homology modeling

Human, yeast and *P. falciparum* subunit beta-5 sequences were aligned using ClustalW. The published crystal structure of the yeast proteosome in complex with salinosporamide A [25] was examined and the residues proximal to the inhibitor binding site were identified. Substitutions identified were subsequently considered for the possible effect on inhibitor binding between species.

### In vivo Studies

Swiss-Webster female mice weighing 25–30 grams (Hilltop Lab Animals, Scottdale, PA) were inoculated intraperitoneal (IP) with 5×10^6^ *P. yoelii* (yoelii yoelii) parasites and separated into three groups of five mice (day 1). On days 2 (24 hours after parasite challenged) through 4 groups were treated by IP injection of salinosporamide A (99% purity) at 130 µg/kg, 13 µg/kg and 0 µg/kg (using a volume of 100 µl for each concentration) or days 1, 2 and 4 by oral route at 250 µg/kg, 130 µg/kg and 0 µg/kg. In a third experiment two groups of five mice were challenge on days 1, 2 and 4 by subcutaneous injection of salinosporamide A at 130 µg/kg and 0 µg/kg. [Salinosporamide A was made fresh on treatment days, dissolved in 100% DMSO and serially diluted with 5% Solutol (Solutol HS 15; polyethylene glycol 660 12-hydroxystearate; BASF, Shrevport, LA) yielding a final concentration of 2% DMSO. The vehicle control (0 µg/kg) consisted of 2% DMSO and 98% (5% solutol) [22]]. Parasitemia was traced daily by light microscopic analysis of giemsa stained thin smears and confirmed by flow cytometry analysis. Blood samples of 4 µL were collected and fixed in 1mL of 0.25% glutaraldehyde (Polysciences) in PBS (pH 7.4), stained with Hoechst 33342 (Invitrogen Molecular Probes), and the average of 20,000 events collected on a Becton Dickson FACSAria.
